# Combination immunotherapy and active-specific tumor cell vaccination augments anti-cancer immunity in a mouse model of gastric cancer

**DOI:** 10.1186/1479-5876-9-140

**Published:** 2011-08-22

**Authors:** Natasja K van den Engel, Dominik Rüttinger, Margareta Rusan, Robert Kammerer, Wolfgang Zimmermann, Rudolf A Hatz, Hauke Winter

**Affiliations:** 1 Department of Surgery, Klinikum Grosshadern, Ludwig-Maximilians-University, Munich, Germany; 2 Institute of Immunology, Friedrich-Loeffler-Institut, Tübingen, Germany; 3 Tumor Immunology Laboratory, LIFE-Center, Klinikum Grosshadern, Ludwig-Maximilians-University, Munich, Germany

## Abstract

**Background:**

Active-specific immunotherapy used as an adjuvant therapeutic strategy is rather unexplored for cancers with poorly characterized tumor antigens like gastric cancer. The aim of this study was to augment a therapeutic immune response to a low immunogenic tumor cell line derived from a spontaneous gastric tumor of a CEA424-SV40 large T antigen (CEA424-SV40 TAg) transgenic mouse.

**Methods:**

Mice were treated with a lymphodepleting dose of cyclophosphamide prior to reconstitution with syngeneic spleen cells and vaccination with a whole tumor cell vaccine combined with GM-CSF (a treatment strategy abbreviated as LRAST). Anti-tumor activity to subcutaneous tumor challenge was examined in a prophylactic as well as a therapeutic setting and compared to corresponding controls.

**Results:**

LRAST enhances tumor-specific T cell responses and efficiently inhibits growth of subsequent transplanted tumor cells. In addition, LRAST tended to slow down growth of established tumors. The improved anti-tumor immune response was accompanied by a transient decrease in the frequency and absolute number of CD4^+^CD25^+^FoxP3^+ ^T cells (Tregs).

**Conclusions:**

Our data support the concept that whole tumor cell vaccination in a lymphodepleted and reconstituted host in combination with GM-CSF induces therapeutic tumor-specific T cells. However, the long-term efficacy of the treatment may be dampened by the recurrence of Tregs. Strategies to counteract suppressive immune mechanisms are required to further evaluate this therapeutic vaccination protocol.

## Background

Gastric cancer is a common disease in industrial countries and is associated with a poor prognosis. Over 50 percent of potentially curatively operated gastric cancer patients relapse within 5 years. Subsequent chemo- or radiation therapy is mostly insufficient [1]. Therefore, the development of new adjuvant treatments with a favorable "therapeutic index", (i.e., good tolerability and demonstrated anti-tumor activity), are desperately needed. Active-specific immunotherapy (i.e., therapeutic vaccination) may represent such an option.

Active-specific immunotherapy aims to improve the patient's ability to mount a therapeutic immune response against cancer. Nevertheless, inducing an immune response against the tumor is by itself not sufficient, and clinical results with cancer vaccines have been sobering [2], even though the first therapeutic vaccine based on autologous dendritic cells (DCs) called Provenge (sipuleucel-T, Dendreon Corp., Seattle, WA, USA) was recently approved for the treatment of hormone refractory prostate cancer [3]. Few vaccination studies in patients with gastric cancer have been published, which demonstrated antibody responses or peptide-specific IFN-γ responses and cytotoxicity by isolated cytotoxic T cells, but did not show strong clinical responses [4-6].

To increase the frequency of circulating tumor-specific T cells is likely to be one important minimal requirement for a successful therapy [7]. To obtain sufficient expansion of such lymphocytes, several therapeutic strategies have been adopted, including prior lymphodepleting, non-myeloablative chemotherapy with cyclophosphamide followed by reconstitution of the lymphocyte pool by infusion of autologous immune cells [8-10]. Lymphopenia naturally induces a proliferative response to maintain homeostasis [11,12]. This stimulates antigen-specific T cells directed towards antigens contained in the tumor vaccine. In preclinical models of melanoma, this strategy increased the frequency of tumor-specific T cells in tumor vaccine-draining lymph nodes (TVDLN) extensively and enhanced the therapeutic efficacy of active-specific and adoptive immunotherapy strategies [13-15]. In addition to lymphopenia-induced proliferation, the elimination of regulatory T cells (Treg) and the creation of a beneficial host microenvironment by affecting components of the innate immune system are alternatively proposed as immunomodulatory effects of preparative chemotherapy with e.g. cyclophosphamide [16-18].

A recently introduced strategy to increase the therapeutic efficacy of tumor vaccination is to combine different immunological approaches, i) applying multifaceted antigen vaccines to target a broad spectrum of tumor antigens, ii) providing co-stimulation, iii) reducing or eliminating suppressive immune cells, e.g. Tregs [7], and iv) blocking tumor-induced immune suppression mediated by e.g. TGF-β [19]. Such a multifactorial vaccination approach may be especially suitable for tumor entities that exhibit a low immunogenicity, as has been described for gastric cancer [20]. Only a few tumor-associated antigens, mostly so-called cancer testis antigens, have been identified to be expressed in gastric tumors [21-23], but this has not yet resulted in successful therapeutic approaches targeting these antigens [24].

In order to explore novel therapeutic vaccination strategies for gastric cancer, we have established cell lines from the spontaneously growing gastric tumors of CEA424-SV40 TAg transgenic mice [25,26]. In the current study, we aimed to enhance the therapeutic anti-tumor immunity in a subcutaneous mouse model of gastric cancer by (i) combining a low immunogenic whole tumor cell vaccine (prepared from the established gastric cell lines) with granulocyte macrophage colony-stimulating factor (GM-CSF) to stimulate local antigen presentation and by (ii) pretreatment with cyclophosphamide to enhance proliferation of tumor-specific T cells and to reduce the frequency of Tregs. Here, we show that lymphodepletion by preparative treatment with cyclophosphamide followed by reconstitution with naïve spleen cells enhances the anti-tumor immunity induced by a whole cell vaccine. This treatment strategy, LRAST, induced a long-term anti-tumor immune response against subsequent tumor challenge and tended to slow down growth of established tumors. GM-CSF significantly reinforced the tumor-specific immune response induced by the tumor vaccine. Furthermore, we observed a transient reduction of Tregs, supporting the priming of a tumor-specific immune response.

## Methods

### Mouse strains and cell lines

C57BL/6 mice were obtained from Charles River (Sulzfeld, Germany). Mice were bred and kept under standard pathogen-free conditions in the animal facility of the Walter-Brendel Center, Ludwig-Maximilians-University of Munich. The animal experiments were performed after approval by the local regulatory agency (Regierung von Oberbayern, Munich, Germany). For tumorigenicity and immunogenicity assays female mice were used at 8-12 weeks of age. The gastric cancer cell lines mGC8 and 424GC were established previously from gastric tumors which developed spontaneously in CEA424-SV40 TAg-transgenic mice (C57BL/6-Tg(CEACAM5-Tag)L5496Wzm) [25,26]. The MCA 310 fibro sarcoma cell line was kindly provided by Dr. B.A. Fox (Portland, OR). Gastric cancer cell lines were cultured in RPMI1640 supplemented with 10% fetal calf serum (FCS "Gold"; PAA Laboratories, Coelbe, Germany), 2 mM L-glutamine, non-essential amino acids and 1 mM sodium pyruvate (Invitrogen, Karlsruhe, Germany). For culturing MCA 310 tumor cells and *in vitro *assays, the medium was supplemented with 10% FCS from Invitrogen (complete medium, CM).

### Tumor cell vaccination (prophylactic/therapeutic), LRAST

To determine the immunogenicity of the tumor cells, 10^7 ^tumor cells were irradiated with 10,000 rad and subcutaneously injected into mice. Two weeks later, the mice were challenged by subcutaneous injection of 3 × 10^6 ^viable tumor cells into the opposite flank. Experimental groups generally consisted of 5 mice. Tumor development was followed by serial measurements of the tumor diameter and is depicted as tumor size (mm^2^) = d × D, where d and D were the shortest and the longest tumor diameter, respectively. Animals were euthanized when D reached 10 mm. Lymphopenia was induced by i.p. injection of cyclophosphamide (Cytoxan, 200 mg/kg; Baxter, Halle, Germany). This dose was chosen since earlier studies have shown an increased proliferation and long-term survival of antigen-specific T cells at this dose of cyclophosphamide, alone or in combination with fludarabine [18,27]. After 24 h, mice were reconstituted with 2 × 10^7 ^naïve syngeneic splenocytes followed by s.c. vaccination with irradiated mGC8 cells (10^7^, 10,000 rad) with or without a s.c. injection of GM-CSF (1 μg, Peprotech, Rocky Hill, NJ) diluted in HBSS and emulsified with an equal volume of incomplete Freund's adjuvant (IFA; Sigma-Aldrich, Taufkirchen, Germany) as described elsewhere [28], to induce an active-specific immune response. Naïve, non-lymphopenic mice served as control. In order to treat established s.c. tumors (therapeutic setting), viable mGC8 cells (10^6^) were injected 4 days before vaccination and tumor vaccinations were repeated every two weeks for a total of 4 vaccinations.

### *In vitro *T cell activation and expansion

For T cell analyses, mice were vaccinated by s.c. injection with 1.2 × 10^7 ^live mGC8 tumor cells on four sites, near the extremities (3 × 10^6 ^per injection). Where indicated, lymphodepletion and reconstitution were performed as described above and GM-CSF/IFA was applied at all four vaccine sites (0.25 μg per injection). TVDLNs were harvested nine days after vaccination and lymph node cells were polyclonally activated with an anti-CD3 monoclonal antibody (mAb; 5 μg/ml, 2C11, kindly provided by Dr. H.M. Hu, Portland, OR) for 2 days at 2 × 10^6 ^cells/ml in CM in 24-well plates. Subsequently cells were expanded at 2 × 10^5 ^cells/ml in CM supplemented with 60 IU/ml of interleukin-2 (IL-2, Proleukin, Chiron, Ratingen, Germany) for 4 days. After 4 days, cytokine release assays were performed as described elsewhere [29] with the following modifications: T cells (10^6 ^cells) were washed and cultured alone or stimulated with tumor cells (0.2 × 10^6 ^cells), or immobilized anti-CD3 antibody in 1 ml of CM supplemented with gentamycin (Lonza, Cologne, Germany) and 60 IU IL-2/ml in a 48-well tissue culture plate at 37°C, 5% CO_2 _for 18 h. The tumor targets included the tumor cell line used for vaccination (mGC8) and a related gastric tumor cell line (424GC). An unrelated, syngeneic tumor cell line (MCA 310) served as a negative control. Supernatants were analyzed by ELISA. TAg-specific peptides T1 and T2 were previously described [30] and added in a final concentration of 10 μg/ml.

### Cell-mediated cytotoxicity assay

Cell-mediated lysis was determined using standard 4-h ^51^Cr-release assays [31]. Cryopreserved TVDLN cells were thawed, stimulated with anti-CD3 for 2 days and IL-2 for 4 days according to the protocol used for the cytokine release assay. Na_2_(^51^Cr)O_4 _(NEN, Boston, MA)-labeled target cells (2000 per well) were incubated with stimulated effector cells for 4 hours at indicated effector-to-target cell ratios in complete medium in round bottom 96-well tissue culture plates. Spontaneous release was determined by incubating target cells alone; total release was determined by directly counting labeled cells. Percentage cytotoxicity was calculated as follows: percentage specific lysis = [experimental counts per minutes (cpm) - spontaneous cpm/total cpm - spontaneous cpm] × 100. Duplicate measurements were done in all experiments.

### ELISA

For capture and detection of IFN-γ in supernatants by conventional sandwich ELISA, we used mAb R4-6A2 and biotinylated mAb XMG1.2, respectively (BD Biosciences, Heidelberg, Germany). Anti-IL-5 antibodies were purchased from R&D Systems (Wiesbaden-Nordenstadt, Germany). Supernatants were analyzed in duplicate. Extinction was analyzed at 405/490 nm on a TECAN microplate ELISA reader (TECAN, Crailsheim, Germany) with the EasyWin software (TECAN). The detection limit of the ELISA for IFN-γ was 125 pg/ml.

### White blood cell count

To determine the degree of lymphopenia induced by cyclophosphamide treatment, 10 μl of blood were drawn from the tail vein into heparinized capillaries at different time points. The blood was diluted 1:10 in Türk's solution (Merck, Darmstadt, Germany) and the white blood cells (WBC) were counted using light-microscopy.

### Flow cytometry

For surface staining cells were washed with PBS and suspended in PBS supplemented with 0.5% (w/v) bovine serum albumin (BSA) and 0.02% (w/v) sodium azide. Non-specific binding of antibodies to Fc receptors was blocked by preincubation of the cells with rat anti-mouse CD16/CD32 monoclonal antibody 2.4G2 (1 μg/10^6 ^cells, BD Biosciences) for 15 min. Subsequently the cells were incubated with the mAb of interest for 30 min at 4°C, washed and analyzed using a FACScan (BD Biosciences). Dead cells were excluded by propidium iodide staining. Collected data were analyzed using the Cell Quest Pro software (Version 4.0.2). The following reagents and mAbs against murine antigens from BD Biosciences were used: phycoerythrin (PE)-conjugated anti-mouse CD11b, PE-conjugated anti-mouse CD4, PE-conjugated anti-mouse CD8 and fluorescein isothiocyanate (FITC)-conjugated anti-mouse Gr1 mAb (RB6-8C5; Ly-6G, Ly6C). Allophycocyanin (APC)-conjugated anti-mouse CD25 mAb was obtained from Invitrogen. For staining of intracellular Foxp3, a FITC-conjugated antibody and buffers were purchased from eBiosciences (San Diego, CA, USA) and staining was performed according to the manufacturer's instructions.

### Statistical analysis

Survival curves for tumor-free survival were plotted according to the Kaplan-Meier method and were compared using the log-rank test. Cytokine responses are presented as mean +/- SE. They were analyzed using a one way analysis of variance (ANOVA) with a Newman-Keuls post hoc test. Tumor sizes were analyzed using the Mann-Whitney-U test. Differences in expression of cellular markers as measured by flow cytometry were compared using the Student's *t *test. Statistical analyses were performed using GraphPad Prism software. For all analyses, *p *values below 0.05 were considered to be significant.

## Results

### Active-specific tumor cell vaccination alone mostly fails to induce a protective immune response

**Figure 1:**
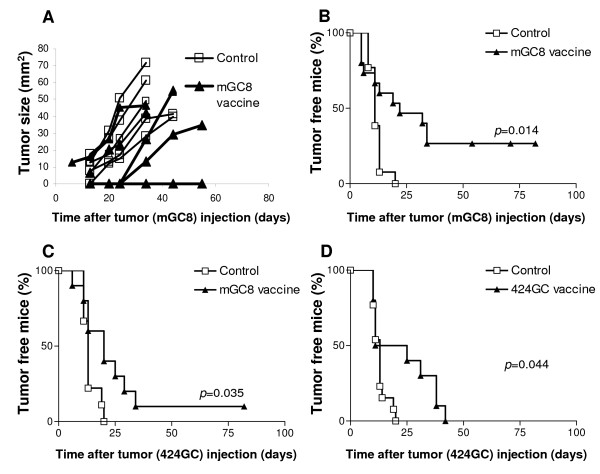
**Determination of the immunogenicity of the gastric tumor cell lines mGC8 and 424GC**. Mice were vaccinated s.c. with 10^7 ^irradiated tumor cells. After 2 weeks, vaccinated and control mice were s.c. injected with 3 × 10^6 ^viable tumor cells and tumor growth was monitored. (A) Development of s.c. tumors after vaccination and challenge with mGC8 cells. Representative result of one of three independent experiments is shown. Each line represents a single mouse (n = 5). (B) Tumor-free survival as observed after treatment as described in A; sum of three independent experiments; vaccine group n = 15, control group n = 13. (C) Tumor-free survival following vaccination with mGC8 and challenge with 424GC cells, sum of two independent experiments (n = 10; control group n = 9). (D) Tumor-free survival after vaccination and challenge with 424GC, sum of two independent experiments (n = 10; control group n = 13).

To study novel strategies for immunotherapy of gastric cancer, we previously established the gastric cancer cell lines mGC8 and 424GC from CEA424-SV40 TAg-transgenic C57BL/6 mice [25]. These cell lines express epithelial cell markers and form tumors in 100% of mice when transplanted subcutaneously (s.c.) at 300,000 cells per injection into C57BL/6 mice [25]. To test the immunogenicity of the cell lines, C57BL/6 mice were vaccinated s.c. with 10^7 ^irradiated mGC8 cells and challenged two weeks later with a single s.c injection of 3 × 10^6 ^live mGC8 cells. In the majority of the immunized mice, tumor growth progressed similar to the control group (Figure 1A). Only four of fifteen (27%) vaccinated mice were completely protected against a subsequent tumor challenge during the observation period of 55 days (Figure 1B). None of the control mice without vaccination was protected and their s.c. tumors were detectable within 20 days after tumor challenge.

In further experiments, we tested the potential of the mGC8 vaccine to induce cross-protection against the syngeneic gastric tumor 424GC. One of ten vaccinated mice (10%) was protected after challenge with live 424GC cells, indicating some cross-reactivity between these tumor cell lines (Figure 1C). In contrast, vaccination with irradiated 424GC cells failed to induce protection against challenge with 424GC cells (Figure 1D). However, a delay in tumor growth was observed in 50% of the mice. Based on these data we concluded that the cell line mGC8 does exhibit low immunogenicity and we hypothesized that under optimized conditions mGC8 may have the potential to induce a protective immune response.

### LRAST enhances anti-tumor immunity induced by tumor cell vaccination resulting in a long-term protection against s.c. tumor challenge

To optimize therapeutic efficacy of the mGC8 tumor cell vaccine we administered the vaccine during lymphopenia-induced T cell proliferation combined with GM-CSF to stimulate local antigen presentation. First, we determined whether cyclophosphamide (200 mg/kg, i.p.) followed by reconstitution with syngeneic splenocytes (LP) had the desired effect on white blood cell depletion and recovery. A single i.p. injection of cyclophosphamide caused lymphopenia in the peripheral blood within one day. The lymphopenia was obvious until day 4, confirming the findings in peripheral blood and spleens in other studies [16,32]. Peripheral leukocyte cell numbers recovered within 9 days (Additional file 1, Figure S1). The tumor vaccine was applied early in the immune recovery phase in order to create optimal conditions for the induction of a systemic immune response against tumor antigens during homeostatic proliferation.

**Figure 2:**
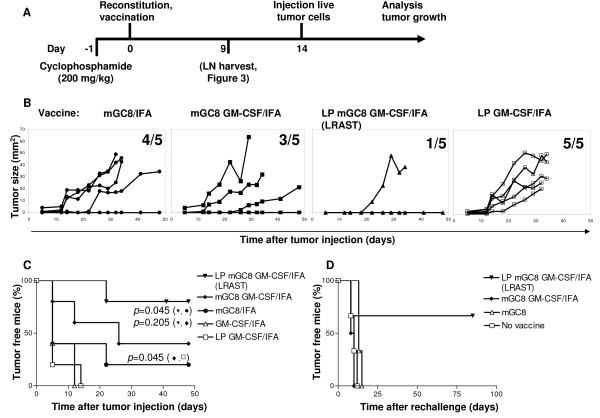
**Improved efficacy of the mGC8 tumor cell vaccine when combined with lymphopenia and reconstitution**. (A) LRAST treatment schema. One day after lymphopenia induction (cyclophosphamide, 200 mg/kg, i.p.), C57BL/6 mice were reconstituted by i.v. injection with 2 × 10^7 ^splenocytes from naïve mice and vaccinated s.c. with 10^7 ^irradiated mGC8 cells and GM-CSF/IFA. Two weeks after vaccination, mice were challenged with 3 × 10^6 ^live mGC8 tumor cells and tumor growth was monitored. (B) Subcutaneous tumor growth of mice vaccinated with mGC8/IFA alone, with mGC8 and GM-CSF/IFA, with mGC8 and GM-CSF/IFA after induction of lymphopenia and reconstitution with spleen cells (LRAST), or the latter treatment without tumor vaccination (LP + GM-CSF/IFA) (n = 5 per group). The number of mice that developed a subcutaneous tumor within 50 days is indicated per group. (C) Tumor-free survival of the groups described in B and of another control group without tumor vaccination: GM-CSF/IFA. Tumor-free survival of LRAST-treated mice was significantly improved compared with mice vaccinated with mGC8 alone (*p *= 0.045). Tumor-free survival of LRAST- and mGC8 GM-CSF/IFA- treated groups was significantly different from the control group LP GM-CSF/IFA (*p *= 0.002 and p = 0.045, respectively), (n = 5 per group). (D) Tumor-free survival of all protected mice from experiment 2B/2C after rechallenge with s.c. injection of 3 × 10^6 ^live mGC8 cells at day 60 and of a new control group without vaccination. The data also include two protected mice of Figure 1B that were rechallenged with live mGC8 at day 80 after mGC8 vaccination. (LRAST, n = 3; mGC8 GM-CSF/IFA, n = 2; mGC8, n = 3; no vaccine, n = 3). LP, induction of lymphopenia followed by reconstitution with spleen cells.

To further enhance the induction of tumor-specific T cells, vaccines are generally combined with adjuvants like GM-CSF, KLH or CpG [33-36]. Gene-modified tumor cells that continuously secrete low levels of GM-CSF have been successfully used to generate effective immune responses [37,38]. In order to mimic the continuous GM-CSF secretion without the necessity to genetically modify the tumor cells, we mixed GM-CSF with IFA to get a creamy emulsion. This emulsion was injected s.c., adjacent to the vaccine site. To investigate the impact of lymphopenia driven proliferation, we compared s.c. tumor growth in mice after vaccination with either mGC8 alone or mGC8 combined with an injection of GM-CSF in IFA, or the latter vaccination following treatment with cyclophosphamide and reconstitution with naïve splenocytes (LRAST, Figure 2A). Although vaccination with mGC8 GM-CSF/IFA without lymphodepletion seemed to delay s.c. tumor growth when compared to the mGC8 vaccination alone, the overall protective effect was low with 3 of 5 and 4 of 5 mice developing s.c. tumors within 50 days, respectively (Figure 2B). In contrast, induction of lymphopenia followed by reconstitution with naïve splenocytes and mGC8 vaccination in the presence of GM-CSF (LRAST) clearly improved the protective effect of the vaccination with only one of five mice developing a s.c. tumor (Figure 2B). In contrast, lymphodepletion, reconstitution and GM-CSF/IFA alone without tumor vaccination was not protective since all mice developed a s.c. tumor (Figure 2B). The percentage of tumor-free mice was significantly increased in the LRAST group (80%) as compared to the group vaccinated with mGC8 alone (20%), *p *= 0.045 (Figure 2C). The tumor-free survival of mice treated with mGC8 GM-CSF/IFA was significantly enhanced compared to LP GM-CSF/IFA-treated mice (*p *= 0.045), indicating the necessity of the tumor cells in the LRAST treatment.

In order to determine whether the protected (tumor-free) mice had developed a systemic, long-term anti-tumor immunity, we injected live mGC8 tumor cells into the flank opposite to the first tumor injection site at day 60. Only mice treated with LRAST (2 out of 3) showed complete protection during the observation period of 3 months after the rechallenge (66%, Figure 2D), suggesting the induction of a long-term protective immune response in these mice. Tumor-free mice of the treatment groups without lymphodepletion developed s.c. tumors within 12 days after rechallenge, which was comparable to the tumor development in control mice that had not been vaccinated (Figure 2D).

### Increased tumor-specific IFN-γ release and cell-mediated cytotoxicity by tumor vaccine-draining lymph node (TVDLN) cells after vaccination with mGC8 cells and GM-CSF/IFA

**Figure 3:**
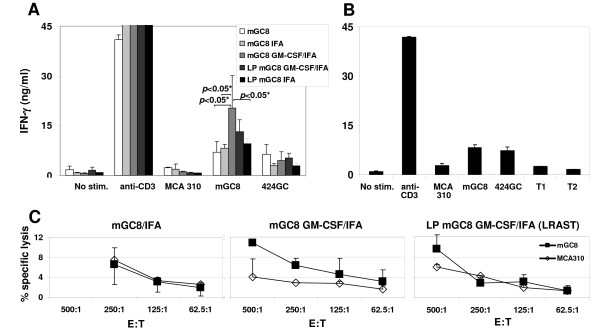
**Tumor-specific IFN-γ release and cell-mediated cytotoxicity after vaccination with mGC8 cells and GM-CSF**. T cells generated from TVDLN at day nine after vaccination were polyclonally activated and expanded as described in the Methods section and tested for tumor-specific IFN-γ release and cell-mediated cytotoxicity. In the cytokine release assay, T cells were either cultured alone, with an anti-CD3 antibody, with a syngeneic but unrelated tumor, MCA 310, with the related tumor cells 424GC or with mGC8 cells. Supernatants were harvested 18 h later for quantification of IFN-γ (and IL-5, not shown) by ELISA. (A) Vaccination with mGC8 with or without LP, GM-CSF, and IFA. Data are presented as the mean of two independent experiments in which co-cultures were performed in duplicate (± SE). IFN-γ secretion was significantly increased in the mGC8 GM-CSF/IFA group (*p *< 0.05) compared with the mGC8-, mGC8 IFA-, and LP mGC8 IFA-groups. LP, induction of lymphopenia followed by reconstitution. (B) Vaccination with mGC8 cells; TVDLN were additionally co-cultured with the TAg peptides T1 and T2. Means of duplicate measurements and SE are indicated (n = 4 for tumor cell lines and the non-stimulated control). (C) Cytotoxicity of TVDLN against mGC8 (black symbols) and MCA310 (open symbols) at declining effector-to-target cell ratio (E:T). Means of duplicate measurements (+/- AVEDEV) are shown. The experiment was repeated after restimulation of the LN cells with irradiated mGC8 tumor cells (10:1) followed by 5 days culture in CM supplemented with 60 IU/ml IL-2 revealing similar results (not shown). AVEDEV: average of the absolute deviations of the numbers above from their mean.

We hypothesized that the mice in the LRAST group would harbor more tumor-specific T cells in their tumor vaccine-draining lymph nodes as compared to mice treated with the mGC8 vaccine alone. To compare the effect of the different treatment strategies on the generation of tumor-specific T cells, TVDLN cells were isolated nine days after vaccination (Figure 2A) and analyzed in a cytokine release assay. While cytokine responses after restimulation with the syngeneic unrelated tumor cell line MCA 310 were low, all vaccinated mice showed release of IFN-γ, but not IL-5 after restimulation with mGC8 and 424GC tumor cells (Figure 3A and not shown, respectively). Addition of IFA to the mGC8 vaccine did not change the tumor-specific IFN-γ release of the TVDLN cells, however, lymphodepletion tended to increase tumor-specific IFN-γ release (Figure 3A). Significant increase of IFN-γ secretion was detected in the group that was vaccinated with mGC8 GM-CSF/IFA compared with the control group that was vaccinated with mGC8 alone, the group vaccinated with mGC8 IFA as well as the lymphodepleted group that was vaccinated with mGC8 IFA (*p *< 0.05), but not compared with the LRAST-treated group (LP mGC8 GM-CSF/IFA). Hence, GM-CSF seemed to be the main factor that caused significant enhancement of the tumor-specific immune response induced by the tumor vaccine. However, GM-CSF alone could not improve the mGC8 vaccine to induce a significant and durable protective anti-tumor immune response *in vivo *(Figure 2D).

To determine whether the tumor-specific IFN-γ release mainly resulted from a response to the TAg, which is a foreign protein in C57BL/6 mice, we restimulated TVDLN from mice vaccinated with mGC8 with the TAg-specific peptides T1 and T2. IFN-γ release by TVDLN cells restimulated with T1 or T2 was not above the levels produced by non-stimulated or MCA 310-stimulated cells and was therefore not tumor specific (Figure 3B).

From three groups, isolated TVDLN cells were abundant and could be cryopreserved to test for cytotoxicity at a later time point. Cells from mGC8 IFA-treated mice demonstrated non-specific lysis since cytotoxicity occurred in mGC8 cells and MCA310 cells to a similar level (Figure 3C). In contrast, LN cells from mGC8 GM-CSF/IFA-treated mice induced specific lysis of mGC8 cells at an E:T ratio of 500:1 and 250:1. The specific lysis of mGC8 cells by LN cells from LRAST-treated mice at an E:T ratio of 500:1 did not appear to be significantly different from that of MCA310 cells in a repeated experiment. Thus, the cytotoxicity data confirm the results of the IFN-γ release assay in that cells from mGC8 GM-CSF/IFA-treated mice show the highest secretion of IFN-γ and the highest specific lysis.

### LRAST potentially also impacts tumor growth of established s.c. tumors

**Figure 4:**
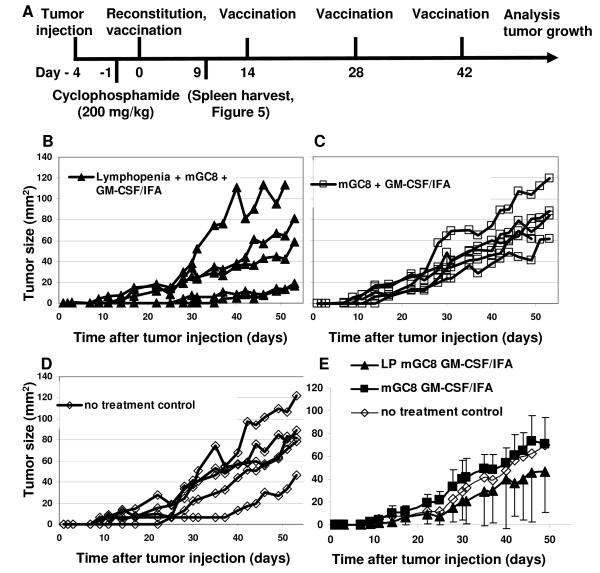
**Effect of LRAST on tumor growth in mice with established tumors**. (A) LRAST treatment schema in a therapeutic setting. C57BL/6 mice received a s.c. injection with 10^6 ^viable mGC8 tumor cells. Three days later, mice in the LRAST group were treated with cyclophosphamide and were reconstituted with spleen cells 24 h later. The same day (day 0), mice were vaccinated with irradiated mGC8 cells (10^7^) and injected with GM-CSF in IFA. One group received no vaccination (no treatment control). The vaccinations with mGC8 and GM-CSF/IFA were repeated every other week for a total of four vaccinations. Tumor growth curves are shown for the individual mice in (B) the LRAST group (n = 5), (C) the mGC8 GM-CSF/IFA-vaccinated group, without cyclophosphamide and reconstitution (n = 5), and (D) the no treatment control group (n = 5). (E) Mean tumor sizes per group shown in B, C and D are plotted (+/- SEM), n = 5 per group. Cyclophosphamide pretreatment tended to delay tumor growth.

After identifying LRAST as an effective treatment to protect against s.c. growing gastric tumors (prophylactic setting), we determined the efficacy of this strategy against the growth of 3-days established s.c. tumors (therapeutic setting, Figure 4A). In the LRAST-treated group, two of five mice showed a clear delay in s.c. tumor development (Figure 4B). In the group treated without cyclophosphamide (mGC8, GM-CSF/IFA) all tumors developed without delay (Figure 4C). Similar tumor growth was seen in the no treatment control (Figure 4D). Thus, although the mean growth of the s.c. tumors was not significantly different between the treatment groups, LRAST tended to delay tumor growth of established s.c. tumors (Figure 4E). Since the mGC8 tumor cells originate from gastric tumors, which developed spontaneously in CEA424-SV40 TAg-transgenic mice, we tested in a pilot experiment whether our vaccination strategy inhibits the spontaneous development of these gastric tumors and thus affects the survival of the transgenic mice. Treatment was started when the mice were 8 weeks of age (n = 6) and weight loss was used as a surrogate marker for the development of the gastric tumor. Mice rapidly lost weight between 95 and 105 days of age and we detected no difference between vaccinated mice and untreated controls (data not shown).

### The efficacy of LRAST is accompanied by a decrease of Tregs

**Figure 5:**
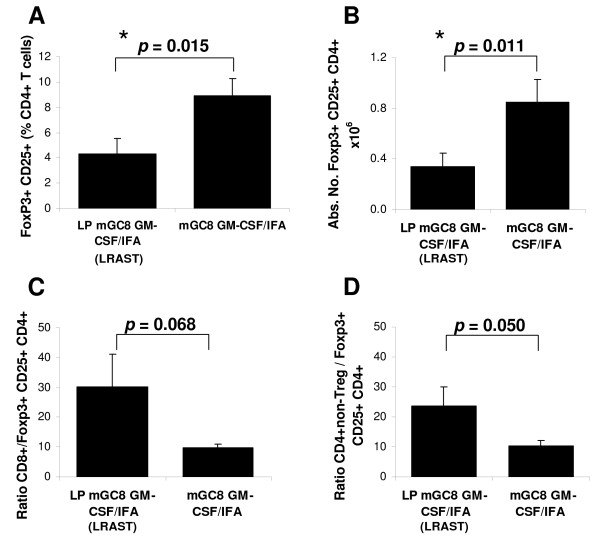
**Effect of LRAST on the frequency of CD4^+^CD25^+^Foxp3^+ ^cells**. Mice were treated with LRAST or mGC8 GM-CSF/IFA in a therapeutic setting as described in Figure 4A. The mice were killed at day 9 after vaccination and splenocytes were analyzed by flow cytometry for the expression of Treg markers (FoxP3 and CD25). (A) Percentage of FoxP3^+^CD25^+ ^cells calculated as a percentage of CD4^+ ^T cells. (B) Absolute number of CD4^+^CD25^+^Foxp3^+ ^cells calculated from initial splenocyte counts. (C) Ratio of CD8^+ ^T cells to CD4^+^CD25^+^Foxp3^+ ^(Tregs) and (D) Ratio of CD4^+ ^non-Tregs to Tregs. (LRAST, n = 4; mGC8 GM-CSF/IFA, n = 2). Means and SE are indicated.

Several publications report on a decrease in regulatory T cells in spleens and lymph nodes (defined as CD4^+ ^CD25^+ ^cells) and subsequent enhancement of the anti-tumor response when including cyclophosphamide in an immunotherapeutic strategy [16,17]. We analyzed splenocytes from mice in the LRAST group and in the group treated with mGC8 GM-CSF/IFA without lymphodepletion for the presence of CD4^+ ^CD25^+ ^FoxP3^+ ^cells (referred to as Tregs). All mice had 3-days established s.c. tumors at treatment start and were analyzed at day 9 after tumor challenge (Figure 4A). Spleen cells from LRAST mice revealed a 2-fold decrease in the frequency of CD4^+ ^CD25^+ ^FoxP3^+ ^cells compared with vaccinated mice without lymphodepletion (Figure 5A). Similarly, the absolute number of CD4^+ ^CD25^+ ^FoxP3^+ ^cells was significantly lower in LRAST mice (Figure 5B). As a consequence the ratio of CD8^+ ^T cells to CD4^+ ^CD25^+ ^FoxP3^+ ^Tregs and the ratio of CD4^+ ^non Tregs to CD4^+ ^CD25^+ ^FoxP3^+ ^Tregs were increased in LRAST-treated mice (Figure 5C and 5D). The decrease of Tregs appeared to be transient since analysis of splenocytes two months after therapy start showed an increased frequency of CD4^+ ^CD25^+ ^Foxp3^+ ^Tregs in LRAST-treated mice similar to the frequency detected in mGC8 GM-CSF/IFA-treated mice and control mice without vaccination (data not shown).

As has been published before, cyclophosphamide treatment can lead to an increase in Gr1^+^CD11b^+ ^myeloid-derived suppressor-like cells (MDSC) in de spleen [18]. We detected a more than 10-fold increase in the frequency Gr1^+^CD11b^+ ^cells in LRAST mice compared with mGC8 GM-CSF/IFA-treated mice at day 9 after vaccination, but they decreased to similar frequencies as in control mice without vaccination at two months after start of the treatment (data not shown).

## Discussion

Several reports have shown that active-specific tumor vaccination administered to a lymphopenic host may result in significantly enhanced anti-tumor immune responses [8,13]. Meanwhile, this study design has been translated into early phase clinical trials for several tumor entities [7,9]. However, there are neither preclinical nor clinical studies that address this therapeutic strategy in gastric cancer. The goal of active-specific tumor vaccination is to induce a systemic tumor-specific immune response especially against low- or non-immunogenic tumors. The aim of this study was to increase the therapeutic efficacy of a vaccination with the low immunogenic gastric tumor cell line mGC8. Consistent with previous reports on other tumor entities [8,15,39], we demonstrate here for the first time that the treatment with cyclophosphamide prior to tumor vaccination in the presence of GM-CSF can efficiently induce long-term protection against subcutaneous tumor growth in a gastric cancer model.

In earlier publications, tumor cell lines genetically modified to secrete GM-CSF or other immunostimulatory cytokines were compared with regard to their effectiveness as a cancer vaccine [37,40]. GM-CSF-secreting tumor vaccines appeared to be most potent to induce long-lasting tumor-specific immunity and have been used in clinical studies [41,42]. Due to the presence of GM-CSF at the vaccine site, antigen-presenting cells (APC) are recruited, activated and capable of activating tumor-specific T cells in the vaccine-draining lymph nodes [33,37]. A future aim of our immunotherapeutic approaches is to use autologous tumor samples for vaccination instead of cell lines. Since gene transfer into freshly derived tumor cells is laborious and may not be very efficient [43], we aimed to apply GM-CSF separately to the tumor cells. The easiest way to do this would be the co-administration of recombinant GM-CSF to the irradiated tumor cells. However, this would require frequent applications of the cytokine due to its short half-life *in vivo *[44], and would probably yield less potent anti-tumor responses compared to GM-CSF secreting cells [33,45]. Approaches that encapsulate or modify GM-CSF to provide sustained release locally at the vaccine site have been shown to result in anti-tumor immune responses comparable to that of GM-CSF-secreting tumor cells [44,46]. In addition, emulsions with IFA have been described to induce a strong and long-term immune response and were suggested to be stable for a few weeks [47,48]. Therefore, we emulsified GM-CSF in IFA and we applied the emulsion subcutaneously at the vaccine site in order to enhance the immune response. Indeed, we found that application of emulsified GM-CSF, but not IFA alone, during vaccination increased the induction of tumor-specific T cells as measured by tumor-specific IFN-γ release from TVDLN cells. In addition, mice vaccinated with irradiated tumor cells in the presence of GM-CSF/IFA showed a significant enhancement of tumor-free survival as compared to lymphodepleted mice treated with GM-CSF/IFA without the tumor vaccine. This indicates the necessity of the presence of tumor antigens for successful LRAST treatment.

While low doses of GM-CSF as an adjuvant have been described to increase vaccine-induced immune responses (reviewed in [49]), in our model the induction of a long-term therapeutic immune response *in vivo *resulted only from the combination of cyclophosphamide treatment with GM-CSF application and not from GM-CSF alone. This emphasizes the expected potency of lymphodepletion applied prior to vaccination to enhance the therapeutic efficacy of a vaccination.

Unexpectedly, application of cyclophosphamide and reconstitution with naïve syngeneic splenocytes prior to the tumor vaccination with GM-CSF (LRAST) did not further increase but rather tended to decrease the tumor-specific immune response *in vitro *as determined by tumor-specific IFN-γ secretion and specific lysis of mGC8 tumor cells by TVDLN cells. This discrepancy between *in vitro *and *in vivo *observations may in part be explained by the fact that significantly less T cells could be recovered from TVDLN following LRAST as compared to TVDLN from other treatment groups. It is conceivable that the remaining LN cells may be more sensitive towards further handling than LN cells that were not affected by cyclophosphamide and that therefore the results do not reflect *in vivo *CTL activity in our setting. On the other hand, the *in vivo *CTL response may be influenced by other mechanisms, e.g. Treg, which do not necessarily have an inhibitory effect when studying CTL activity *in vitro*. Since the mGC8 GM-CSF/IFA-treated group shows a higher number of Treg than the LRAST group, it is conceivable the *in vivo *anti-tumor response is suppressed in the former group.

At least two mechanisms have been proposed for the positive effect of cyclophosphamide pre-treatment on tumor vaccination: (i) increased homeostatic expansion of antigen-specific T cells in a lymphopenic environment and (ii) depletion of regulatory T cells. We addressed the first mechanism by analyzing the tumor-specific cytokine release in T cells isolated from TVDLN 9 days after vaccination. TVDLN cells from LRAST-treated and LP mGC8 IFA-treated mice tended to secrete increased levels of tumor-specific IFN-γ compared with TVDLN cells from control mice. Considering the enhancement of anti-tumor immunity after the LRAST treatment, one may anticipate that an augmented secretion of IFN-γ reflects an increase in the number of tumor-specific T cells in the LRAST-treated mice. However, alternatively an increase in cytokine expression per cell may have occurred as well. A preliminary ELISpot analysis suggested that TVDLN from LRAST-treated mice had both a larger number of IFN-γ producing cells and released more tumor specific IFN-γ per cell as compared to control mice (not shown).

Several studies have reported on a depletion of Tregs as another mechanism to explain the beneficial effect of cyclophosphamide treatment [8,16-18]. Tregs are known to efficiently down-modulate immune responses and depletion of these cells has been shown to enhance the anti-tumor immune response in various tumor models [50,51]. Consistent with other reports, we observed a rapid decline in white blood cells one day after a single i.p. application of cyclophosphamide and a gradual recovery of the cell numbers during the following week [32]. Although the absolute numbers of lymphocytes in the peripheral blood normalized after 9 days (Additional file 1, Figure S1), the frequency and the absolute number of FoxP3^+ ^CD25^+ ^CD4^+ ^Treg cells were decreased in the spleen of LRAST-treated mice as compared to vaccinated mice without lymphodepletion (Figure 5A and 5B). This is consistent with previous findings that describe a transient reduction of Tregs in the spleens of mice in the first 10 days after cyclophosphamide (100 mg/kg) treatment [16]. In that study, in addition to a reduction of CD4^+^CD25^+ ^cells after cyclophosphamide treatment, a loss of *FoxP3 *and *GITR *gene expression as well as a reduction of Treg function was reported. In our experiments, the decline in the number of Tregs, the increase in the ratio of CD8^+ ^T cells to FoxP3^+ ^CD25^+ ^CD4^+ ^Tregs and the lymphopenic environment after cyclophosphamide treatment favor enhanced priming of tumor-specific immune responses during vaccination. This is consistent with the efficacy of the LRAST treatment against s.c. tumor growth *in vivo *(Figure 2). The precise role of Treg in the induction of anti-tumor immunity is subject of planned investigations in our laboratory and will be analyzed by depletion of Treg from the cell population used for reconstitution as well as by adoptive transfer of Treg after cyclophosphamide treatment.

In the experiments using a therapeutic setting we aimed to boost the tumor-specific immune response by giving repeated vaccinations. Although some mice in the LRAST group showed benefit by displaying a delayed tumor growth, the mean growth was not significantly different from the group without cyclophosphamide treatment. We observed that approximately two months after LRAST treatment, the proportion of FoxP3^+^CD25^+^CD4^+ ^T cells had increased again to the frequencies of the other treatment groups without lymphodepletion. Thus, it seems that an initial decrease in Tregs after vaccination was followed by a secondary "induction" of Tregs. Interestingly, we also observed higher numbers of FoxP3^+^CD25^+^CD4^+ ^T cells in mice that showed a long-term protective response after LRAST (data not shown). Therefore, we assume that a later increase of Tregs does not necessarily affect the anticancer effect of the treatment. It remains to be determined whether late appearance of Tregs actually has an impact on the therapeutic efficacy of the overall anti-tumor response. A recent study reported that the use of multiple vaccinations had a negative effect on the generation of therapeutic effector T cells [52]. The authors showed that multiple vaccinations increased the absolute number of CD4^+^Foxp3^+ ^Tregs in the peripheral blood and in the spleens, which decreased the therapeutic efficacy of splenocytes when adoptively transferred into tumor-bearing mice. In support of these results, we have recently observed that repeated vaccination with irradiated autologous tumor vaccines did not maintain a long-term reduction of Foxp3^+ ^Tregs in the peripheral blood of non-small cell lung cancer patients after lymphodepleting chemotherapy (Van den Engel et al., manuscript in preparation).

Consistent with a previous report [18], we detected high numbers of CD11b^+^Gr1^+ ^cells in the spleen 9 days after pretreatment with cyclophosphamide. This increase in Gr1^+^CD11b^+ ^cells in cyclophosphamide-treated mice suggests the presence of myeloid-derived suppressor cells that could limit the immune response, as has been suggested in several reports [53,54]. In contrast, other reports suggest a beneficial effect through inhibition of tumor growth by the MDSC [18,55]. It remains to be determined whether these cells have inhibitory influence on the immune response that is elicited by LRAST.

Recently, a related s.c. gastric cancer mouse model was used to test the therapeutic efficacy of a dendritic cell vaccine loaded with irradiated gastric tumor cells in combination with CpG oligonucleotides [56]. In that study, tumor cells from the cell line mGC3 were used as the antigen source in the DC vaccine. The cell lines mGC3 and mGC8 were established from CEA424-SV40 TAg tumors and both cell lines display similar expression levels of epithelial cell surface markers, MHC class I molecules and the large-T antigen [25], which suggests that they may exhibit comparable therapeutic potential. Indeed, prophylactic vaccination with the DC vaccine improved survival in wild type mice injected with mGC3 tumor cells and caused long-term protection, similarly to our results with LRAST using the cell line mGC8. However, neither active immunization using the DC tumor cell vaccine nor adoptive transfer of tumor-reactive splenocytes did change survival of transgenic CEA424-SV40 TAg mice developing spontaneous gastric tumors, suggesting immunological tolerance toward multiple tumor-associated epitopes in these mice [56]. Correspondingly, we did not see a survival benefit in CEA424-SV40 TAg mice treated with LRAST in a pilot experiment (not shown). Therefore, we support the view that developing an immunotherapy, which is clinically effective in these transgenic mice will be challenging and will require additional immune-activating approaches, for example by inactivating cells that suppress immune responses.

## Conclusions

Our data show that induction of lymphopenia, followed by reconstitution with naïve spleen cells and GM-CSF application during vaccination leads to a sustained protection against gastric tumors. We observed that this approach (LRAST) increases the systemic anti-tumor immune response and initially reduces the number of FoxP3^+^CD25^+^CD4^+ ^Tregs. Induction of regulatory cellular mechanisms like MDSC and recurrence of Tregs may, in turn, dampen the therapeutic efficacy of LRAST on the long term. Modulation or depletion of the suppressive cell populations may be a promising way to further improve the therapeutic strategy of LRAST.

## List of abbreviations

LRAST: lymphopenia, reconstitution and active-specific tumor cell vaccination; GM-CSF: granulocyte macrophage colony-stimulating factor; IFA: incomplete Freund's adjuvant; mAb: monoclonal antibody; DC: dendritic cell; LP: induction of lymphopenia followed by reconstitution with spleen cells; Tregs: regulatory T cells; MDSC: myeloid-derived suppressor cells; TVDLN: tumor vaccine-draining lymph node.

## Competing interests

The authors declare that they have no competing interests.

## Authors' contributions

NKE and HW designed the animal experiments. DR provided support, discussed the data and reviewed the manuscript. NKE and MR planned and conducted the experiments and discussed the data. NKE coordinated the study and drafted the manuscript. HW discussed the data and reviewed the manuscript. RK established the cell lines and participated in coordination and design of the initial experiments. WZ participated in design of initial experiments and reviewed the manuscript. RH directed the laboratory where the studies were performed, participated in experimental design and obtained support for the project. All authors read and approved the final manuscript.

## Supplementary Material

Additional file 1 **Figure S1 Changes in WBC count after induction of lymphopenia with cyclophosphamide**. Mice were treated with cyclophosphamide at day 0 (200 mg/kg, i.p.). After 24 h, mice were reconstituted with 2 × 10^7 ^naïve syngeneic splenocytes. The control group did neither receive cyclophosphamide nor splenocytes. WBC were counted at day 0, 1 (before reconstitution), 4 and 9; n = 5 per group. *p < 0.05, using Student's *t*-test. Click here for file

## References

[B1] Catalano V Labianca R Beretta GD Gatta G de Braud F Van Cutsem E Gastric cancer Crit Rev Oncol Hematol 2005 54 209 241 10.1016/j.critrevonc.2005.01.002 15890270

[B2] Rosenberg SA Yang JC Restifo NP Cancer immunotherapy: moving beyond current vaccines Nat Med 2004 10 909 915 10.1038/nm1100 15340416 PMC1435696

[B3] Kantoff PW Higano CS Shore ND Berger ER Small EJ Penson DF Redfern CH Ferrari AC Dreicer R Sims RB Xu Y Frohlich MW Schellhammer PF Sipuleucel-T immunotherapy for castration-resistant prostate cancer N Engl J Med 2010 363 411 422 10.1056/NEJMoa1001294 20818862

[B4] Kono K Takahashi A Sugai H Fujii H Choudhury AR Kiessling R Matsumoto Y Dendritic cells pulsed with HER-2/neu-derived peptides can induce specific T-cell responses in patients with gastric cancer Clin Cancer Res 2002 8 3394 3400 12429626

[B5] Gilliam AD Watson SA Henwood M McKenzie AJ Humphreys JE Elder J Iftikhar SY Welch N Fielding J Broome P Michaeli D A phase II study of G17DT in gastric carcinoma Eur J Surg Oncol 2004 30 536 543 10.1016/j.ejso.2004.03.009 15135483

[B6] Sato Y Fujiwara T Mine T Shomura H Homma S Maeda Y Tokunaga N Ikeda Y Ishihara Y Yamada A Tanaka N Itoh K Harada M Todo S Immunological evaluation of personalized peptide vaccination in combination with a 5-fluorouracil derivative (TS-1) for advanced gastric or colorectal carcinoma patients Cancer Sci 2007 98 1113 1119 10.1111/j.1349-7006.2007.00498.x 17459063 PMC11158036

[B7] Poehlein CH Ruttinger D Ma J Hu HM Urba WJ Fox BA Immunotherapy for melanoma: the good, the bad, and the future Curr Oncol Rep 2005 7 383 392 10.1007/s11912-005-0066-1 16091201

[B8] Machiels JP Reilly RT Emens LA Ercolini AM Lei RY Weintraub D Okoye FI Jaffee EM Cyclophosphamide, doxorubicin, and paclitaxel enhance the antitumor immune response of granulocyte/macrophage-colony stimulating factor-secreting whole-cell vaccines in HER-2/neu tolerized mice Cancer Res 2001 61 3689 3697 11325840

[B9] Ruttinger D van den Engel NK Winter H Schlemmer M Pohla H Grutzner S Wagner B Schendel DJ Fox BA Jauch KW Hatz RA Adjuvant therapeutic vaccination in patients with non-small cell lung cancer made lymphopenic and reconstituted with autologous PBMC: first clinical experience and evidence of an immune response J Transl Med 2007 5 43 10.1186/1479-5876-5-43 17868452 PMC2020458

[B10] Eto M Kamiryo Y Takeuchi A Harano M Tatsugami K Harada M Kiyoshima K Hamaguchi M Teshima T Tsuneyoshi M Yoshikai Y Naito S Posttransplant administration of cyclophosphamide and donor lymphocyte infusion induces potent antitumor immunity to solid tumor Clin Cancer Res 2008 14 2833 2840 10.1158/1078-0432.CCR-07-1742 18451251

[B11] Cho BK Rao VP Ge Q Eisen HN Chen J Homeostasis-stimulated proliferation drives naive T cells to differentiate directly into memory T cells J Exp Med 2000 192 549 556 10.1084/jem.192.4.549 10952724 PMC2193235

[B12] Mackall CL Hakim FT Gress RE Restoration of T-cell homeostasis after T-cell depletion Semin Immunol 1997 9 339 346 10.1006/smim.1997.0091 9405262

[B13] Hu HM Poehlein CH Urba WJ Fox BA Development of antitumor immune responses in reconstituted lymphopenic hosts Cancer Res 2002 62 3914 3919 12124318

[B14] Ma J Urba WJ Si L Wang Y Fox BA Hu HM Anti-tumor T cell response and protective immunity in mice that received sublethal irradiation and immune reconstitution Eur J Immunol 2003 33 2123 2132 10.1002/eji.200324034 12884286

[B15] Ma J Poehlein CH Jensen SM LaCelle MG Moudgil TM Ruttinger D Haley D Goldstein MJ Smith JW Curti B Ross H Walker E Hu HM Urba WJ Fox BA Manipulating the host response to autologous tumour vaccines Dev Biol (Basel) 2004 116 93 107 15603186

[B16] Lutsiak ME Semnani RT De Pascalis R Kashmiri SV Schlom J Sabzevari H Inhibition of CD4(+)25+ T regulatory cell function implicated in enhanced immune response by low-dose cyclophosphamide Blood 2005 105 2862 2868 10.1182/blood-2004-06-2410 15591121

[B17] Ghiringhelli F Larmonier N Schmitt E Parcellier A Cathelin D Garrido C Chauffert B Solary E Bonnotte B Martin F CD4+CD25+ regulatory T cells suppress tumor immunity but are sensitive to cyclophosphamide which allows immunotherapy of established tumors to be curative Eur J Immunol 2004 34 336 344 10.1002/eji.200324181 14768038

[B18] Salem ML Kadima AN El Naggar SA Rubinstein MP Chen Y Gillanders WE Cole DJ Defining the ability of cyclophosphamide preconditioning to enhance the antigen-specific CD8+ T-cell response to peptide vaccination: creation of a beneficial host microenvironment involving type I IFNs and myeloid cells J Immunother 2007 30 40 53 10.1097/01.cji.0000211311.28739.e3 17198082

[B19] Petrausch U Jensen SM Twitty C Poehlein CH Haley DP Walker EB Fox BA Disruption of TGF-beta signaling prevents the generation of tumor-sensitized regulatory T cells and facilitates therapeutic antitumor immunity J Immunol 2009 183 3682 3689 10.4049/jimmunol.0900560 19692636 PMC2850273

[B20] Toge T Effectiveness of immunochemotherapy for gastric cancer: a review of the current status Semin Surg Oncol 1999 17 139 143 10.1002/(SICI)1098-2388(199909)17:2<139::AID-SSU9>3.0.CO;2-R 10449686

[B21] Kono K Takahashi A Amemiya H Ichihara F Sugai H Iizuka H Fujii H Matsumoto Y Frequencies of HER-2/neu overexpression relating to HLA haplotype in patients with gastric cancer Int J Cancer 2002 98 216 220 10.1002/ijc.10179 11857411

[B22] Wang Y Wu XJ Zhao AL Yuan YH Chen YT Jungbluth AA Gnjatic S Santiago D Ritter G Chen WF Old LJ Ji JF Cancer/testis antigen expression and autologous humoral immunity to NY-ESO-1 in gastric cancer Cancer Immun 2004 4 11 15516106

[B23] Bolli M Schultz-Thater E Zajac P Guller U Feder C Sanguedolce F Carafa V Terracciano L Hudolin T Spagnoli GC Tornillo L NY-ESO-1/LAGE-1 coexpression with MAGE-A cancer/testis antigens: a tissue microarray study Int J Cancer 2005 115 960 966 10.1002/ijc.20953 15751033

[B24] Chen Y Wu K Guo C Liu C Han S Lin T Ning X Shi R Shi Y Fan D A novel DNA vaccine containing four mimicry epitopes for gastric cancer Cancer Biol Ther 2005 4 308 312 10.4161/cbt.4.3.1502 15876863

[B25] Nockel J van den Engel NK Winter H Hatz RA Zimmermann W Kammerer R Characterization of gastric adenocarcinoma cell lines established from CEA424/SV40 T antigen-transgenic mice with or without a human CEA transgene BMC Cancer 2006 6 57 10.1186/1471-2407-6-57 16536871 PMC1421424

[B26] Thompson J Epting T Schwarzkopf G Singhofen A Eades-Perner AM van der Putten H Zimmermann W A transgenic mouse line that develops early-onset invasive gastric carcinoma provides a model for carcinoembryonic antigen-targeted tumor therapy Int J Cancer 2000 86 863 869 10.1002/(SICI)1097-0215(20000615)86:6<863::AID-IJC16>3.0.CO;2-4 10842202

[B27] Koike N Pilon-Thomas S Mule JJ Nonmyeloablative chemotherapy followed by T-cell adoptive transfer and dendritic cell-based vaccination results in rejection of established melanoma J Immunother 2008 31 402 412 10.1097/CJI.0b013e31816cabbb 18391755

[B28] Vulliet R Improved technique for the preparation of water-in-oil emulsions containing protein antigens Biotechniques 1996 20 797 800 8723921 10.2144/96205bm14

[B29] Meijer SL Dols A Hu HM Chu Y Romero P Urba WJ Fox BA Reduced L-selectin (CD62LLow) expression identifies tumor-specific type 1 T cells from lymph nodes draining an autologous tumor cell vaccine Cell Immunol 2004 227 93 102 10.1016/j.cellimm.2004.01.006 15135291

[B30] Kammerer R Stober D Riedl P Oehninger C Schirmbeck R Reimann J Noncovalent association with stress protein facilitates cross-priming of CD8+ T cells to tumor cell antigens by dendritic cells J Immunol 2002 168 108 117 11751953 10.4049/jimmunol.168.1.108

[B31] Schleypen JS Baur N Kammerer R Nelson PJ Rohrmann K Grone EF Hohenfellner M Haferkamp A Pohla H Schendel DJ Falk CS Noessner E Cytotoxic markers and frequency predict functional capacity of natural killer cells infiltrating renal cell carcinoma Clin Cancer Res 2006 12 718 725 10.1158/1078-0432.CCR-05-0857 16467081

[B32] Salem ML Diaz-Montero CM Al Khami AA El Naggar SA Naga O Montero AJ Khafagy A Cole DJ Recovery from cyclophosphamide-induced lymphopenia results in expansion of immature dendritic cells which can mediate enhanced prime-boost vaccination antitumor responses in vivo when stimulated with the TLR3 agonist poly(I:C) J Immunol 2009 182 2030 2040 10.4049/jimmunol.0801829 19201856 PMC3066095

[B33] Simmons AD Li B Gonzalez-Edick M Lin C Moskalenko M Du T Creson J VanRoey MJ Jooss K GM-CSF-secreting cancer immunotherapies: preclinical analysis of the mechanism of action Cancer Immunol Immunother 2007 56 1653 1665 10.1007/s00262-007-0315-2 17410360 PMC11029840

[B34] Emens LA Asquith JM Leatherman JM Kobrin BJ Petrik S Laiko M Levi J Daphtary MM Biedrzycki B Wolff AC Stearns V Disis ML Ye X Piantadosi S Fetting JH Davidson NE Jaffee EM Timed sequential treatment with cyclophosphamide, doxorubicin, and an allogeneic granulocyte-macrophage colony-stimulating factor-secreting breast tumor vaccine: a chemotherapy dose-ranging factorial study of safety and immune activation J Clin Oncol 2009 27 5911 5918 10.1200/JCO.2009.23.3494 19805669 PMC2793039

[B35] Kafi K Betting DJ Yamada RE Bacica M Steward KK Timmerman JM Maleimide conjugation markedly enhances the immunogenicity of both human and murine idiotype-KLH vaccines Mol Immunol 2009 46 448 456 10.1016/j.molimm.2008.10.020 19046770 PMC2768258

[B36] Chamoto K Takeshima T Wakita D Ohkuri T Ashino S Omatsu T Shirato H Kitamura H Togashi Y Nishimura T Combination immunotherapy with radiation and CpG-based tumor vaccination for the eradication of radio- and immuno-resistant lung carcinoma cells Cancer Sci 2009 100 934 939 10.1111/j.1349-7006.2009.01114.x 19245434 PMC11158651

[B37] Dranoff G Jaffee E Lazenby A Golumbek P Levitsky H Brose K Jackson V Hamada H Pardoll D Mulligan RC Vaccination with irradiated tumor cells engineered to secrete murine granulocyte-macrophage colony-stimulating factor stimulates potent, specific, and long-lasting anti-tumor immunity Proc Natl Acad Sci USA 1993 90 3539 3543 10.1073/pnas.90.8.3539 8097319 PMC46336

[B38] Arca MJ Krauss JC Aruga A Cameron MJ Shu S Chang AE Therapeutic efficacy of T cells derived from lymph nodes draining a poorly immunogenic tumor transduced to secrete granulocyte-macrophage colony-stimulating factor Cancer Gene Ther 1996 3 39 47 8785710

[B39] Wada S Yoshimura K Hipkiss EL Harris TJ Yen HR Goldberg MV Grosso JF Getnet D Demarzo AM Netto GJ Anders R Pardoll DM Drake CG Cyclophosphamide augments antitumor immunity: studies in an autochthonous prostate cancer model Cancer Res 2009 69 4309 4318 10.1158/0008-5472.CAN-08-4102 19435909 PMC3084614

[B40] Lipshy KA Kostuchenko PJ Hamad GG Bland CE Barrett SK Bear HD Sensitizing T-lymphocytes for adoptive immunotherapy by vaccination with wild-type or cytokine gene-transduced melanoma Ann Surg Oncol 1997 4 334 341 10.1007/BF02303584 9181234

[B41] Soiffer R Hodi FS Haluska F Jung K Gillessen S Singer S Tanabe K Duda R Mentzer S Jaklitsch M Bueno R Clift S Hardy S Neuberg D Mulligan R Webb I Mihm M Dranoff G Vaccination with irradiated, autologous melanoma cells engineered to secrete granulocyte-macrophage colony-stimulating factor by adenoviral-mediated gene transfer augments antitumor immunity in patients with metastatic melanoma J Clin Oncol 2003 21 3343 3350 10.1200/JCO.2003.07.005 12947071

[B42] Nemunaitis J Jahan T Ross H Sterman D Richards D Fox B Jablons D Aimi J Lin A Hege K Phase 1/2 trial of autologous tumor mixed with an allogeneic GVAX vaccine in advanced-stage non-small-cell lung cancer Cancer Gene Ther 2006 13 555 562 10.1038/sj.cgt.7700922 16410826

[B43] Miller DG Adam MA Miller AD Gene transfer by retrovirus vectors occurs only in cells that are actively replicating at the time of infection Mol Cell Biol 1990 10 4239 4242 2370865 10.1128/mcb.10.8.4239-4242.1990 PMC360961

[B44] Daro E Pulendran B Brasel K Teepe M Pettit D Lynch DH Vremec D Robb L Shortman K McKenna HJ Maliszewski CR Maraskovsky E Polyethylene glycol-modified GM-CSF expands CD11b(high)CD11c(high) but notCD11b(low)CD11c(high) murine dendritic cells in vivo: a comparative analysis with Flt3 ligand J Immunol 2000 165 49 58 10861034 10.4049/jimmunol.165.1.49

[B45] Shi FS Weber S Gan J Rakhmilevich AL Mahvi DM Granulocyte-macrophage colony-stimulating factor (GM-CSF) secreted by cDNA-transfected tumor cells induces a more potent antitumor response than exogenous GM-CSF Cancer Gene Ther 1999 6 81 88 10.1038/sj.cgt.7700012 10078967

[B46] Golumbek PT Azhari R Jaffee EM Levitsky HI Lazenby A Leong K Pardoll DM Controlled release, biodegradable cytokine depots: a new approach in cancer vaccine design Cancer Res 1993 53 5841 5844 8261390

[B47] Aucouturier J Dupuis L Ganne V Adjuvants designed for veterinary and human vaccines Vaccine 2001 19 2666 2672 10.1016/S0264-410X(00)00498-9 11257407

[B48] Moncada C Torres V Israel Y Simple method for the preparation of antigen emulsions for immunization J Immunol Methods 1993 162 133 140 10.1016/0022-1759(93)90415-4 8509648

[B49] Parmiani G Castelli C Pilla L Santinami M Colombo MP Rivoltini L Opposite immune functions of GM-CSF administered as vaccine adjuvant in cancer patients Ann Oncol 2007 18 226 232 17116643 10.1093/annonc/mdl158

[B50] Shimizu J Yamazaki S Sakaguchi S Induction of tumor immunity by removing CD25+CD4+ T cells: a common basis between tumor immunity and autoimmunity J Immunol 1999 163 5211 5218 10553041

[B51] Li J Hu P Khawli LA Epstein AL Complete regression of experimental solid tumors by combination LEC/chTNT-3 immunotherapy and CD25(+) T-cell depletion Cancer Res 2003 63 8384 8392 14679000

[B52] LaCelle MG Jensen SM Fox BA Partial CD4 depletion reduces regulatory T cells induced by multiple vaccinations and restores therapeutic efficacy Clin Cancer Res 2009 15 6881 6890 10.1158/1078-0432.CCR-09-1113 19903784 PMC2784281

[B53] Angulo I las Heras FG Garcia-Bustos JF Gargallo D Munoz-Fernandez MA Fresno M Nitric oxide-producing CD11b(+)Ly-6G(Gr-1)(+)CD31(ER-MP12)(+) cells in the spleen of cyclophosphamide-treated mice: implications for T-cell responses in immunosuppressed mice Blood 2000 95 212 220 10607705

[B54] Angulo I Jimenez-Diaz MB Garcia-Bustos JF Gargallo D las Heras FG Munoz-Fernandez MA Fresno M Candida albicans infection enhances immunosuppression induced by cyclophosphamide by selective priming of suppressive myeloid progenitors for NO production Cell Immunol 2002 218 46 58 10.1016/S0008-8749(02)00521-X 12470613

[B55] Pelaez B Campillo JA Lopez-Asenjo JA Subiza JL Cyclophosphamide induces the development of early myeloid cells suppressing tumor cell growth by a nitric oxide-dependent mechanism J Immunol 2001 166 6608 6615 11359814 10.4049/jimmunol.166.11.6608

[B56] Bourquin C von der BP Zoglmeier C Anz D Sandholzer N Suhartha N Wurzenberger C Denzel A Kammerer R Zimmermann W Endres S Efficient eradication of subcutaneous but not of autochthonous gastric tumors by adoptive T cell transfer in an SV40 T antigen mouse model J Immunol 2010 185 2580 2588 10.4049/jimmunol.0903231 20644173

