# Supplementary figures and images for: Combination immunotherapy and active-specific tumor cell vaccination augments anti-cancer immunity in a mouse model of gastric cancer
Source: J Transl Med. 2011 Aug 22;9:140. doi: 10.1186/1479-5876-9-140 (PMC3169470; doi:10.1186/1479-5876-9-140)

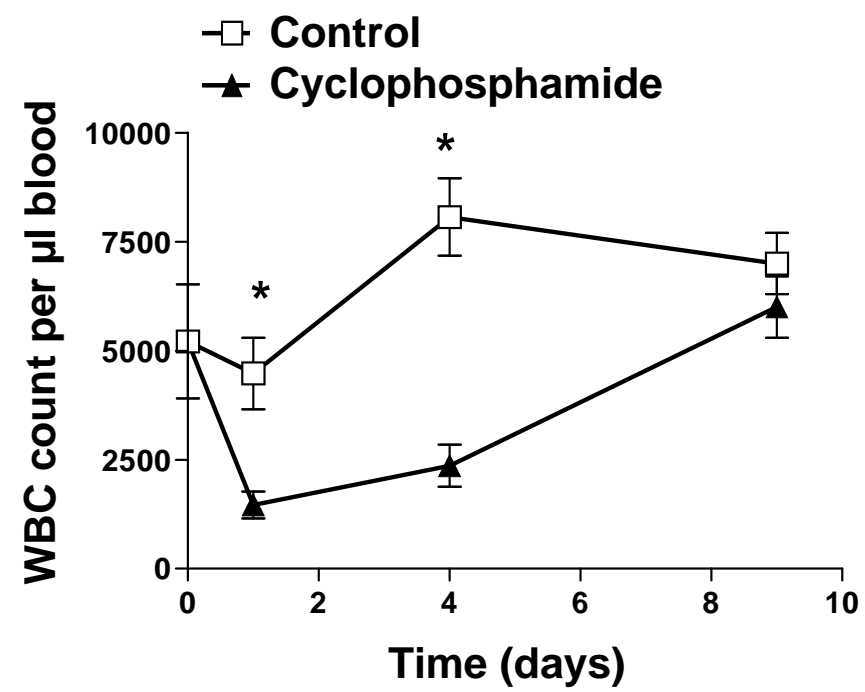

Supplement: Additional file 1 — Figure S1 Changes in WBC count after induction of lymphopenia with cyclophosphamide. Mice were treated with cyclophosphamide at day 0 (200 mg/kg, i.p.). After 24 h, mice were reconstituted with 2 × 10^7^ naïve syngeneic splenocytes. The control group did neither receive cyclophosphamide nor splenocytes. WBC were counted at day 0, 1 (before reconstitution), 4 and 9; n = 5 per group. *p < 0.05, using Student's t-test. [file 1479-5876-9-140-S1.PDF]
